# Relative Contribution of P5 and Hap Surface Proteins to Nontypable *Haemophilus influenzae* Interplay with the Host Upper and Lower Airways

**DOI:** 10.1371/journal.pone.0123154

**Published:** 2015-04-20

**Authors:** Begoña Euba, Javier Moleres, Cristina Viadas, Igor Ruiz de los Mozos, Jaione Valle, José Antonio Bengoechea, Junkal Garmendia

**Affiliations:** 1 Centro de Investigación Biomédica en Red de Enfermedades Respiratorias (CIBERES), Madrid, Spain; 2 Instituto de Agrobiotecnología, CSIC-Universidad Pública Navarra-Gobierno Navarra, Mutilva, Spain; 3 Laboratory Microbial Pathogenesis, Fundación Investigación Sanitaria Illes Balears (FISIB), CSIC-Govern Illes Balears, Bunyola, Spain; 4 Centre for Infection and Immunity, Queen’s University Belfast, Belfast, United Kingdom; Imperial College London, UNITED KINGDOM

## Abstract

Nontypable *Haemophilus influenzae* (NTHi) is a major cause of opportunistic respiratory tract disease, and initiates infection by colonizing the nasopharynx. Bacterial surface proteins play determining roles in the NTHi-airways interplay, but their specific and relative contribution to colonization and infection of the respiratory tract has not been addressed comprehensively. In this study, we focused on the *ompP5* and *hap* genes, present in all *H*. *influenzae* genome sequenced isolates, and encoding the P5 and Hap surface proteins, respectively. We employed isogenic single and double mutants of the *ompP5* and *hap* genes generated in the pathogenic strain NTHi375 to evaluate P5 and Hap contribution to biofilm growth under continuous flow, to NTHi adhesion, and invasion/phagocytosis on nasal, pharyngeal, bronchial, alveolar cultured epithelial cells and alveolar macrophages, and to NTHi murine pulmonary infection. We show that P5 is not required for bacterial biofilm growth, but it is involved in NTHi interplay with respiratory cells and in mouse lung infection. Mechanistically, P5_NTHi375_ is not a ligand for CEACAM1 or α5 integrin receptors. Hap involvement in NTHi375-host interaction was shown to be limited, despite promoting bacterial cell adhesion when expressed in *H*. *influenzae* RdKW20. We also show that Hap does not contribute to bacterial biofilm growth, and that its absence partially restores the deficiency in lung infection observed for the Δ*ompP5* mutant. Altogether, this work frames the relative importance of the P5 and Hap surface proteins in NTHi virulence.

## Introduction

**Fig 1:**
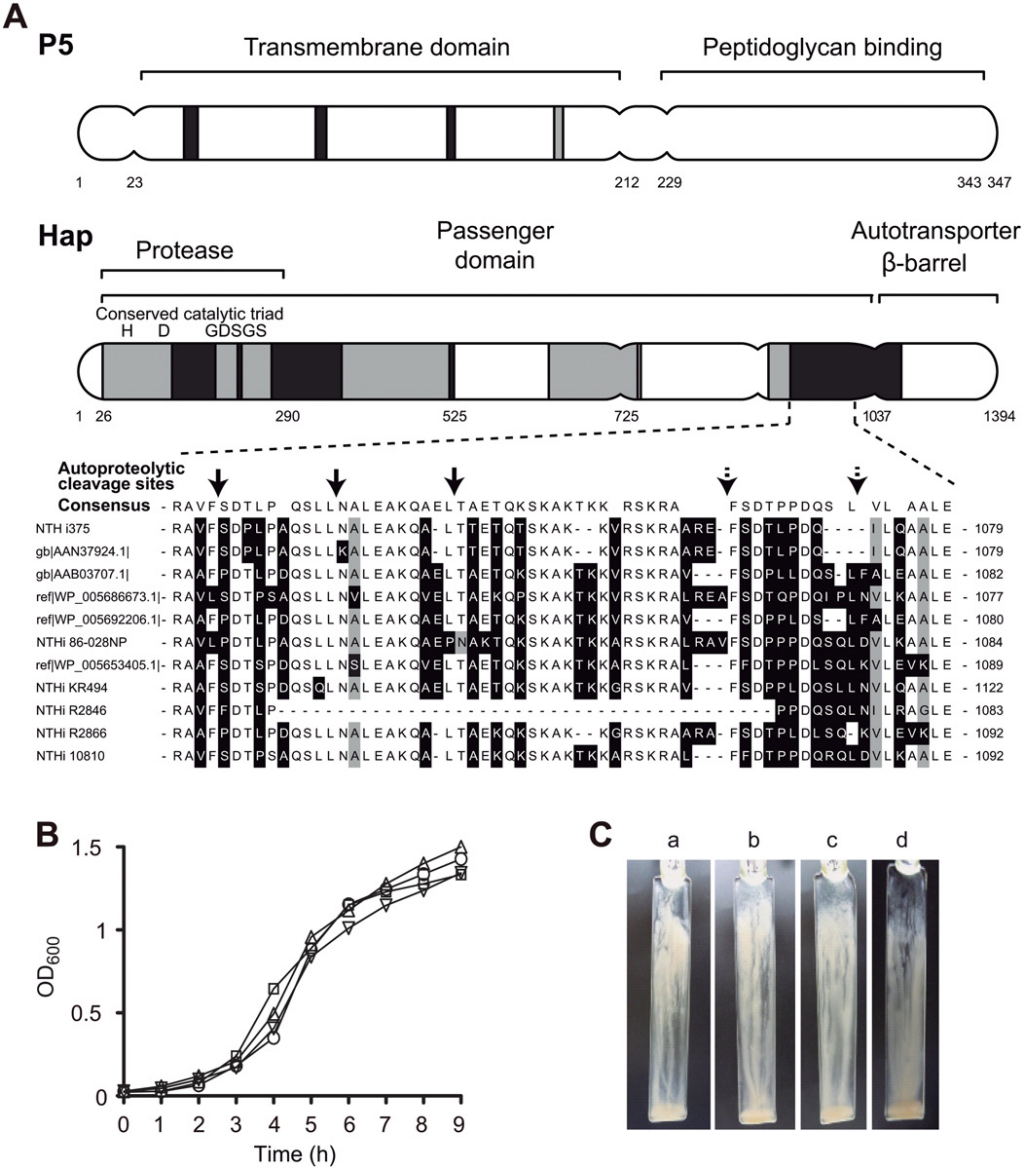
Representation of predicted P5 and Hap structural domains, bacterial growth and biofilm formation. **(A)** Schematic representation of P5 and Hap surface proteins for NTHi375, predicted structural domains and amino acid polymorphisms. Multiple sequence alignments for NTHi375 P5 and Hap were performed in Muscle [49], by using all NCBI available NTHi orthologous proteins. Domain structures were predicted by using the conserved domain database by Marchler-Bauer and co-workers [50], and designated based on previous experimental findings and in agreement with the NCBI conserved domain database. P5 shows four hypervariable regions at the putative surface-exposed loops in the transmembrane domain [5,6,51]. Hap contains a N-terminal signal peptide, a passenger Hap_s_ and a β-barrel Hap_β_ domain. Hap alignment exhibits a hypervariable amino acid mosaic-like pattern [52]. Hap_s_ contains a conserved serine protease domain with the catalytic triad site His_98_, Asp_140_ and Ser_244_ (_242_GDSGS_246_), which mediates autoproteolysis. Predicted cleavage positions are denoted with arrows; dotted arrows show degenerated consensus cleavage sites. Similar residues are represented in white (>90% similarity); variable residues are shaded in gray (>80% similarity); hypervariable residues are shaded in black (<80% similarity). **(B)** Growth curve of NTHi375 wild-type (circle), Δ*ompP5* (triangle), Δ*hap* (square) and Δ*ompP5*Δ*hap* (inverted triangle) strains. **(C)** Biofilm formation under continuous-flow conditions, in microfermenters containing glass slides where bacteria formed the biofilm. Images of a representative experiment for NTHi375 (a), Δ*ompP5* (b), Δ*hap* (c) and Δ*ompP5*Δ*hap* (d) strains.

Nontypable (non-capsulated) *Haemophilus influenzae* (NTHi) is a Gram negative coccobacillus that is a common commensal in the nasopharynx of both children and adults, and also an important cause of localized respiratory tract infections such as acute otitis media, otitis media with effusion, community-acquired pneumonia, and exacerbations of chronic bronchitis and chronic obstructive pulmonary disease (COPD) [1]. Current evidence indicates that NTHi is highly adapted to the human airways [2]. NTHi interplay with host extracellular matrix (ECM) proteins and cell surfaces is facilitated by several proteinaceous adhesins, including the P5 and Hap outer membrane proteins (OMPs) [3]. The *ompP5* and *hap* genes are present in all *H*. *influenzae* isolates sequenced to date [4]. The *ompP5* gene encodes P5, a major outer membrane protein predicted to have hypervariable domains (Fig 1A) [5,6]. P5 has been shown to be an adhesin to human oropharyngeal cells [7], mucin [8], chinchilla eustachian tube mucus [9], and respiratory syncytial virus infected type II pneumocytes [10]. P5 levels seem to be preserved on NTHi biofilms compared to planktonically grown cells [11]. P5 is required for resistance of NTHi to the classical and alternative complement pathways [12], and belongs to a set of virulence genes required in nonvirally infected mice [13]. Depending on the strain, P5 may be important for optimal NTHi growth in rich medium [12], and it may be a bacterial ligand for the carcinoembryonic antigen-related cell adhesion molecule 1 (CEACAM1) [14], playing then a role in nasopharynx colonisation in the chinchilla model [15,16]. NTHi stimulates the expression of intercellular adhesion protein 1 (ICAM-1) on respiratory epithelial cells [17], and P5 has been shown to be a ligand for ICAM-1 [18].

Hap (*Haemophilus* adherence and penetration) is a monomeric self-associating autotransporter (SAAT) with homology to serine-type IgA1 proteases, identified as a bacterial factor promoting interaction with respiratory epithelial cells [3,19]. Hap contains a signal peptide (SP), a passenger domain (Hap_s_) and a β-barrel domain (Hap_β_) [3]. Hap_s_ harbours a C-terminal adhesive region that promotes adherence to human epithelial cells [20], to ECM proteins [21,22], and to neighbouring Hap-expressing bacteria [20], and a N-terminal serine protease domain that modulates Hap autoproteolytic cleavage [23] (Fig 1A). Hap autoproteolytic activity is inhibited by secretory leukocyte protease inhibitor (SLPI), a serine proteinase inhibitor found in human respiratory secretions whose activity results in enhanced Hap adhesive activity [24]. Hap seems to be associated with bacteria within the biofilm, and to be present in the biofilm extracellular matrix [25]. Mutation of a repertoire of genes encoding enzymes involved in the synthesis of the lipooligosaccharide (LOS) core results in decreased *hap* transcription [26].

Despite the wealth of evidence on P5 and Hap, there is not a systematic characterization of the relative contribution of these adhesins to NTHi virulence. In this study, we hypothesised that P5 and Hap may have a differential contribution to NTHi-host interplay through the human respiratory tract. We employed NTHi strain 375 (hereafter NTHi375) to generate single and double mutants defective in the *ompP5* and/or *hap* genes. We evaluated the effect of their disruption on NTHi biofilm growth, on bacterial interaction with a panel of cultured respiratory cells including nasal, pharyngeal, bronchial and alveolar cells, and on mouse respiratory infection. This work allowed us to assign specific roles to P5 and Hap OMPs, providing a context for their relative importance to a range of phenotypic traits and a greater understanding of their contribution to NTHi interplay with the host airways.

## Methods

### Bacterial strains, media and growth conditions

Strains used in this study are described in Table 1. NTHi strains were grown (overnight, 37°C, 5% CO_2_) on Chocolate-agar (Biomérieux) or on brain-heart infusion (BHI)-agar plates supplemented with 10 μg/ml hemin and 10 μg/ml nicotinamide adenine dinucleotide (NAD), referred to as sBHI. NTHi liquid cultures were grown in sBHI (37°C, 5% CO_2_). Erythromycin 10 μg/ml (Erm_10_), kanamycin 11 μg/ml (Km_11_), or chloramphenicol 1 μg/ml (Cm_1_) were used when required. *Escherichia coli* was grown on Luria Bertani (LB) broth or LB-agar plates at 37°C, supplemented with ampicillin 100 μg/ml (Amp_100_), erythromycin 150 μg/ml (Erm_150_), kanamycin 50 μg/ml (Km_50_), or chloramphenicol 30 μg/ml (Cm_30_), when necessary.

**Table 1:** Strains and plasmids used in this study.

Strain	Description	Reference
***H*. *influenzae***		
NTHi375	Wild-type, otitis media clinical isolate	[27]
375Δ*hap*	*hap*::*ermC*, Erm^R^	[29]
375Δ*ompP5*	*ompP5*::*ermC*, Erm^R^	This study
375Δ*hap*Δ*ompP5*	*hap*::*ermC*, *ompP5*::*km*, Erm^R^ Km^R^	This study
RdKW20	Laboratory strain, capsule-deficient serotype d	[44]
RdKW20Δ*ompP5*	*ompP5*::*ermC*, Erm^R^	This study
***E*. *coli***		
CC118	Used for cloning assays	
**Plasmid**		
pGEM-T Easy	Used for cloning assays	Promega
pGEM-T/*ompP5*	*ompP5* from NTHi375 and flanking regions cloned into pGEM-T Easy	This study
pBSLerm	Source of an Erm^R^ cassette	[30]
pUC4K	Source of a Km^R^ cassette	Addgene
pALG-2	pGEM-T/*ompP5* derivative where *ompP5* is disrupted by an Erm^R^ cassette	This study
pALG-3	pGEM-T/*ompP5* derivative where *ompP5* is disrupted by an Km^R^ cassette	This study
pSU20	Cloning vector with a p15A replication origin, Cm^R^	[32]
pSU20- *Pr*::*hap*_*NTHi375*_-HA	pSU20 derivative with a 4.6-kb insert containing *hap* from NTHi375 expressed under its own promoter	This study

To monitor growth, NTHi strains grown on chocolate agar for 24 h were inoculated (2 to 5 colonies) in 20 ml sBHI and incubated for 11 h under shaking. Cultures were then diluted 1:80 in sBHI and incubated for 9 h under the same conditions. OD_600_ was recorded hourly.

NTHi strain 375 (NTHi375) is an OM isolate [4,27,28]; NTHi375Δ*hap* has been described previously [29]. The *ompP5* gene and its respective adjacent regions, was amplified by PCR with *Taq* polymerase (Promega) using NTHi375 genomic DNA as template and primers ompP5-F1 (5´-AGCCAGACTTAATCTATCCGAATAATTTGT)/ompP5-R1 (5´- TTGCGGGTTTTATTTTTCCACTGTGATTAA). The gene-containing fragment was cloned into pGEM-T Easy (Promega), generating pGEMT/ompP5. Cloned PCR product was disrupted by inverse PCR with *Vent* polymerase (New England Biolabs), using primers ompP5-F2 (5´-ACCAATGGCTAACTCGCGTAGGTAAATACC)/ompP5-R2 (5´-CTGCGTATTCTGCACCTACTGCAAATAAAC). An internal 30-bp fragment (nucleotides 521 to 550 in the *ompP5* coding sequence) was replaced by a blunt-ended (excised by *Sma*I) erythromycin resistance cassette from pBSLerm [30], generating plasmid pALG-2 (Table 1). This plasmid was digested with *Not*I to obtain a linear disruption cassette for *ompP5*, that was used to transform NTHi375 using the MIV method [31]. Transformants were screened by plating bacteria on sBHI-agar with Erm_10_, to obtain NTHi375Δ*ompP5*. Same approach and disruption cassette was used to generate *H*. *influenzae* (Hi) RdKW20Δ*ompP5* mutant strain. The NTHi375Δ*ompP5*Δ*hap* double mutant was generated by two successive recombination events. First, strain NTHi375Δ*hap* was generated as described previously [29], and it was then transformed with a disruption cassette for *ompP5*. This cassette was generated by cloning a blunt-ended kanamycin resistance gene released from pUC4K by digestion with *Hinc*II in a vector obtained by inverse PCR of pGEM-T/*ompP5* with primers ompP5-F2/ompP5-R2. The resulting plasmid, pAGL-3, was digested with *Not*I, creating a 3.8 kb linear fragment containing the disruption cassette *ompP5*::*Km* ^*R*^, used to transform NTHi375Δ*hap*. Double recombination events were selected on sBHi-agar with Erm_10_ and Km_11_.

### Expression of Hap_NTHi375_-HA in *H*. *influenzae* RdKW20

The *hap* gene and its upstream 500 bp region, was amplified by PCR with Phusion High-Fidelity DNA Polymerase (Thermo Fisher Scientific) using NTHi375 genomic DNA as template, and primers hap-F1 (5´-ACTATCGTCGTCATTGAACACAATCTTGAT)/hap-R1 (5´-TTAAGCGTAGTCTGGGACGTCGTATGGGTACCAACGATACCCCAATTTCACGCCCAC). The hap-R1 reverse primer was used to introduce an HA tag coding sequence at the 3´end of the *hap* gene. This 4,677 bp blunt PCR product was phosphorylated with T4 kinase (New England Biolabs), and cloned into pSU20 [32], previously digested with *Hinc*II and dephosphorylated with antarctic phosphatase (New England Biolabs), generating pSU20-*Pr*::*hap*_*NTHi375*_-HA. pSU20 and pSU20-*Pr*::*hap*_*NTHi375*_-HA were transformed into electrocompetent Hi RdKW20. Transformations were selected on sBHI-agar with Cm_1_. Hi RdKW20, RdKW20 (pSU20) and RdKW20 (pSU20-*Pr*::*hap*_*NTHi375*_-HA) whole cell extracts were prepared from cultures grown to OD_600_ = 0.9 in sBHI containing Cm_1_, when required. Hap_NTHi375_-HA expression was analyzed by western blot with a primary rabbit anti-HA antibody (Sigma) diluted 1:4000, and a secondary goat anti-rabbit IgG (whole molecule, Sigma) antibody conjugated to horseradish peroxidase, diluted 1:1000.

### Biofilm formation

Biofilm formation under flow conditions was assessed using 60 ml microfermenters (Pasteur Institute, Laboratory of Fermentation) with a continuous flow of medium (40 ml/h) and constant aeration with sterile compressed air (0.3 bar). Submerged Pyrex glass slides served as the growth substratum. Three to four colonies of each strain grown on chocolate-agar were inoculated in 20 ml sBHI and incubated under shaking up to OD_600_ = 1. Approximately 5x10^8^ bacteria from this culture were used to inoculate each microfermenter, which was then run at 37°C for 16 h. The viability on each inoculated bacterial aliquot was tested by serial dilution and plating on sBHI-agar. Biofilm development was recorded with a Nikon Coolpix 950 digital camera. To quantify the biofilm formed, bacteria adhered to the Pyrex slides were resuspended in 10 ml PBS, and OD_600_ of the suspensions was determined. The viability on the bacteria recovered from the biofilm was tested by serial dilution and plating on sBHI-agar. Experiments were performed in duplicate on at least three independent occasions (n≥6).

### Cell culture and bacterial infection

RPMI 2650 human nasal epithelial cells (ATCC CCL-30) were maintained in DMEM with HEPES 10mM, 10% heat inactivated foetal calf serum (FCS) and antibiotics (penicillin and streptomycin) in 75 cm^2^ tissue culture flasks at 37°C with 5% CO_2_. Cells were seeded to 5x10^4^ cells/well in 24-well plates 24 h before infection. Detroit 562 human pharynx epithelium (ATCC CCL-138) was maintained as RPMI 2650 cells, and seeded to 2x10^5^ cells/well 48 h before infection. NCI H-292 mucoepidermoid pulmonary human carcinoma epithelial cells (ATCC CRL-1848) were maintained in RPMI 1640 with Hepes 10mM, 10% FCS and antibiotics, and seeded to 4x10^5^ cells/well 24 h before infection. A549 human carcinomic alveolar basal epithelial cells (ATCC CCL-185) were maintained as described before [33]. Cells were seeded to 6x10^4^ cells/well for 32 h, and serum-starved 16 h before infection. HeLa-BGP is a stably transfected HeLa cell line expressing hCEACAM1-4L receptor [34]. CEACAM1 expression has been previously tested in this cell line, under conditions identical to those used in this study [5]. HeLa-BGP cells were propagated as shown before [34], and seeded to 4x10^5^ cells/well 24 h before infection. Murine alveolar macrophages MH-S (ATCC, CRL-2019) were grown on RPMI 1640 with Hepes 10mM, 10% FCS and antibiotics, and seeded to 7x10^5^ cells/well 16 h before infection.

For NTHi infection, we used previously set up conditions [33,35,36]. RPMI 2650, Detroit 562, NCI H-292, A549, HeLa and HeLa-BGP cells were infected in 1 ml EBSS (Earle's Balanced Salt Solution, Gibco) to get a multiplicity of infection (MOI) of ~100:1. MH-S cells were infected in 1 ml RPMI 1640 with Hepes 10mM and 10% FCS to get a MOI of ~100:1. To monitor adhesion, RPMI 2650, Detroit 562, NCI H-292, A549, HeLa and HeLa-BGP cells were infected for 30 min, and MH-S cells were infected for 1 h. Although this assay does not completely exclude a possible internalization of some bacteria, experimental conditions were previously set to monitor adhesion ([33,36], data not shown). Cells were then washed 5 times with PBS, lysed with 300 μl of PBS-saponin 0.025% for 10 min at room temperature, and serial dilutions were plated on sBHI-agar. For invasion, all epithelial cells were infected for 2 h, washed 3 times with PBS, incubated for 1 h with RPMI 1640 containing 10% FCS, Hepes 10mM and gentamicin 200 μg/ml. For phagocytosis, MH-S cells were infected for 1 h, washed 3 times with PBS, and incubated for 1 h with medium containing gentamicin 300 μg/ml. In all cases, cells were washed 3 times with PBS and lysed as described above. Bacterial adherence to the wells was excluded by monitoring infection of the panel of cell lines by microscopy (data not shown).

When indicated, A549 cells were pretreated for 16 h with 2 μg/ml tunicamycin (Sigma). This treatment did not induce cytotoxicity, verified by measuring the release of lactate dehydrogenase and microscopy (data not shown). Drug exposure was maintained during bacterial contact. Drug exposure had no effect on bacterial viability under the conditions tested (data not shown). α5 integrin analysis was performed by (i) cell incubation with anti-α5 P1D6 (20 μg/ml) function blocking antibody, for 1 h before infection. The antibody was kept during infection; (ii) cell incubation with RGD peptide (10 μM), added to the cells 1 h before infection, and removed before infection. All infections were carried out in triplicate at least three independent times (n>9).

### Mouse assays

These assays were carried out as previously described [29]. CD1 female mice (4 to 5 weeks old) were purchased from Charles River Laboratories (France) and housed under pathogen-free conditions at the Institute of Agrobiotechnology of the Universidad Pública de Navarra (UPNA) facilities (registration number ES/31-2016-000002-CR-SU-US). Animal handling and procedures were in accordance with the current European (Directive 86/609/EEC) and National (Real Decreto 53/2013) legislations, following the FELASA and ARRIVE guidelines, and with the approval of the UPNA Animal Experimentation Committee (Comité de Ética, Experimentación Animal y Bioseguridad-CEEAB, http://www.unavarra.es/invest/comiteEtica.htm) and the local Government authorization. Animal´s condition was monitored daily. For infection, bacteria were recovered with 1 ml PBS from chocolate-agar grown for 16 h, to obtain a suspension of ~5x10^9^ c.f.u./ml. Before infection, mice were anesthetized intraperitoneally with a mixture of ketamine-xylazine (3:1). Each mouse received 20 μl of inoculum (~10^8^ c.f.u.) intranasally. During infection, we did not observe signs of sickness or alterations in behavior, and the use of humane endpoints was not required. Groups of at least 5 mice were euthanized by cervical dislocation and necropsied at selected intervals to determine the number of c.f.u. per lung. Lungs were aseptically removed, individually weighed in sterile bags (Stomacher80, Seward Medical), homogenized, and serially ten-fold diluted in PBS. Each dilution was spread on sBHI-agar (detection limit <10 c.f.u./lung).

### Statistical analysis

For biofilm growth, cell infection and mice infection assays, mean±SD were calculated and statistical comparison of means performed using the two-tail *t* test. In all cases, a p<0.05 value was considered statistically significant. Analyses were performed using Prism software, version 4 for PC (GraphPad Software) statistical package.

## Results

### Construction of NTHi375 mutant strains defective in the surface proteins P5 and Hap

P5 and Hap are two NTHi OMPs encoded by the *ompP5* and *hap* genes, respectively, present in all NTHi isolates analysed to date [4]. In this study, we employed NTHi375, an isolate used for previous studies on NTHi biology and infection [28,29,33], and whose genome sequence is available [37]. We analysed P5 and Hap amino acid sequence on NTHi375, and their level of conservation (Fig 1A, Fig A and Table A in S1 File).

NTHi375 was used to generate NTHi375Δ*ompP5*, NTHi375Δ*hap* [29] and Δ*hap*Δ*ompP5* mutant strains (Table 1). The *ompP5* and *hap* gene expression was monitored by RNA extraction and quantitative real-time PCR on those strains. As expected, we could not detect expression of the *ompP5* gene in NTHi375Δ*ompP5* and NTHi375Δ*hap*Δ*ompP5* strains, neither expression of the *hap* gene in NTHi375Δ*hap* and NTHi375Δ*hap*Δ*ompP5* strains (data not shown). These mutants did not exhibit growth defects compared to the wild-type strain when OD_600_ was monitored over time during growth in sBHI liquid culture (Fig 1B).

### P5 and Hap surface proteins are not required for NTHi biofilm growth

Given that P5 levels have been shown to be preserved on NTHi biofilms [11], and that Hap can be detected in the biofilm extracellular matrix [25], we assessed P5 and Hap contribution to NTHi biofilm growth by using an *in vitro* model system based on the formation of biofilm communities by NTHi375 grown under continuous-flow culture conditions in microfermenters [29,38]. We separately inoculated comparable c.f.u. numbers for each strain, and monitored biofilm development in microfermenters by measuring the turbidity of bacterial suspensions detached from removable glass slides after 16 h. OD_600_ of bacterial suspensions detached from glass slide was similar for the four strains tested, wild-type (OD_600_ = 1.19±0.42), Δ*ompP5* (OD_600_ = 1.26±0.32), Δ*hap* (OD_600_ = 1.24±0.35), and Δ*ompP5*Δ*hap* (OD_600_ = 1.49±0.35). Representative images are shown in Fig 1C. Biofilm viability was quantified by serial dilution plating of bacterial suspensions detached from the removable glass slides, which rendered similar numbers among strains (~10^10^ c.f.u./suspension, i.e. /biofilm).

In sum, under the conditions tested, NTHi375Δ*ompP5*, Δ*hap* and Δ*ompP5*Δ*hap* mutants formed biofilms similar to those observed for the wild-type strain.

### Role of P5 and Hap in NTHi interplay with upper and lower airways epithelial cells

**Fig 2:**
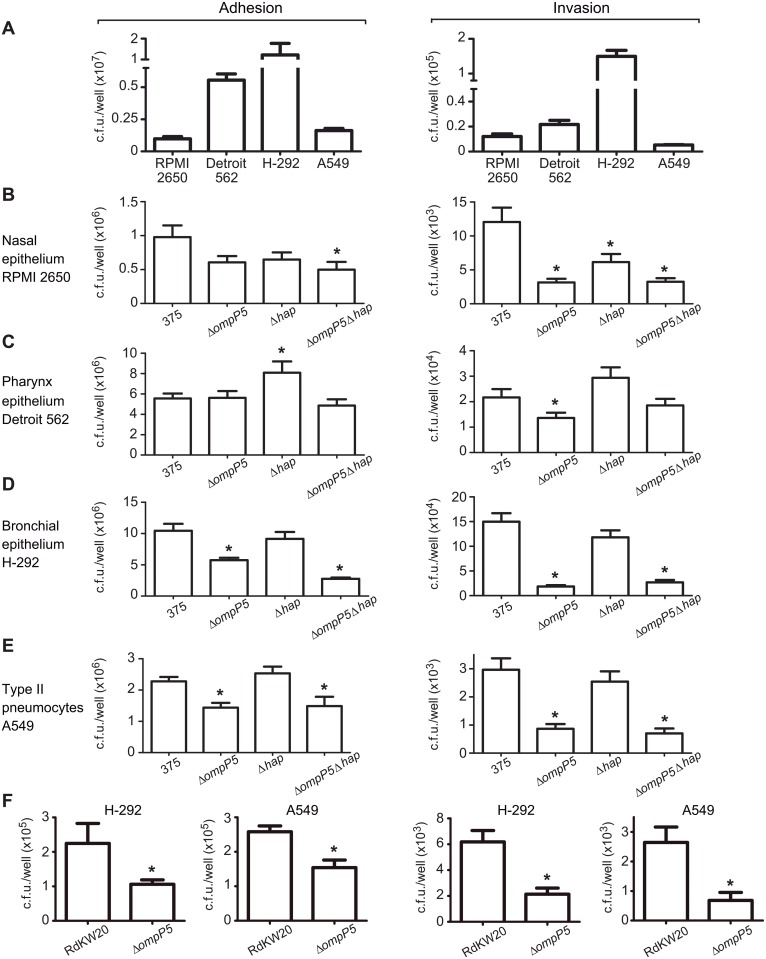
Infection of respiratory epithelial cells by NTHi375 mutant strains lacking the *ompP5* and *hap* genes. Bacterial adhesion is shown in the left and invasion in the right panels, in all sections of this figure. **(A)** NTHi375 interplay with upper and lower human epithelial airways. NTHi375 adhesion to- and invasion of nasal RPMI 2650, pharynx Detroit 562 and bronchial H-292 epithelia and type II pneumocytes A549. Effect of *ompP5* and *hap* gene disruption on NTHi interplay with RPMI 2650 nasal **(B)**, Detroit 562 pharynx **(C)**, NCI H-292 bronchial **(D)**, and A549 type II alveolar **(E)** epithelial cells. NTHi375, Δ*ompP5*, Δ*hap* and Δ*ompP5*Δ*hap* strains were used to assess bacterial adhesion and invasion. **(F)** Effect of *ompP5* gene disruption on Hi RdKW20 interplay with NCI H-292 bronchial and A549 type II alveolar epithelial cells. Hi RdKW20 and Δ*ompP5* mutant strains were used to assess bacterial adhesion and invasion. Experiments were performed in triplicate in at least three independent occasions (n≥9).

To assess the role of P5 and Hap in NTHi interaction with the human airways epithelia, we used nasal RPMI 2650, pharynx Detroit 562, bronchial NCI H-292 and A549 type II pneumocytes cultured epithelial cells. First, we established infection levels for NTHi375 wild-type strain in the four cell types. NTHi375 adhered to the four cell types with variable numbers. Adhesion to nasal cells was significantly lower than that observed for the other cell types tested (mean numbers for RMPI 2650 were lower than those obtained for Detroit 562 and H-292 (p<0.0001), and for A549 (p<0.05) cells); adhesion to Detroit 562 was significantly lower than to NCI H-292 (p<0.0001), but higher than to A549 (p<0.0001) cells; adhesion to NCI H-292 was higher than that observed for the other cell types tested (p<0.0001) (Fig 2A). In terms of NTHi375 internalization into the four cell types, the highest invasion was found for NCI H-292 (p<0.0001) cells, followed by pharynx, nasal and alveolar cells, respectively. Thus, NTHi375 invasion of RMPI 2650 cells was lower than that obtained for Detroit 562 (p<0.05) and H-292 (p<0.0001) cells, and higher than that shown by A549 (p<0.005) cells; invasion of Detroit 562 was lower than that obtained for H-292 (p<0.0001), and higher than the one measured for A549 (p<0.0005) cells (Fig 2A).

Next, we tested the ability of NTHi375Δ*ompP5*, Δ*hap* and Δ*ompP5*Δ*hap* mutants to infect the four cell types. NTHi375Δ*ompP5*, Δ*hap* and Δ*ompP5*Δ*hap* adhesion to RPMI 2650 cells was lower than that displayed by the wild-type strain, although the observed decrease was significant only for NTHi375Δ*ompP5*Δ*hap* (p<0.05). Invasion of the wild-type strain was significantly higher than that observed for Δ*ompP5* (p<0.0005), Δ*hap* (p<0.05) and Δ*ompP5*Δ*hap* (p<0.0005) mutants (Fig 2B). NTHi375Δ*ompP5* and Δ*ompP5*Δ*hap* mutants adhered to Detroit 562 cells at a level similar to the wild-type strain. Conversely, adhesion of NTHi375Δ*hap* to Detroit 562 cells was higher (p<0.05) than that displayed by the wild-type strain. Invasion of NTHi375Δ*ompP5* into Detroit 562 cells was significantly lower (p<0.05) than that displayed by the wild-type strain. NTHi375Δ*hap* and Δ*ompP5*Δ*hap* mutants presented Detroit 562 cell invasion levels similar to the wild-type strain (Fig 2C). We also tested the role of P5 and Hap in NTHi375 interplay with NCI H-292 bronchial cells. Adhesion of NTHi375Δ*ompP5* and Δ*ompP5*Δ*hap*, but not NTHi375Δ*hap*, to NCI H-292 cells was lower than that displayed by the wild-type strain (p<0.005 and p<0.0001, respectively). In agreement with adhesion data, NTHi375Δ*ompP5* and Δ*ompP5*Δ*hap* mutants invaded NCI H-292 cells to a lower extent than the wild-type strain (p<0.0001), but NTHi375Δ*hap* entered NCI H-292 at the same level as the wild-type strain (Fig 2D). Finally, adhesion of NTHi375Δ*ompP5* and Δ*ompP5*Δ*hap* to A549 cells was lower than that displayed by the wild-type strain (p<0.001 and p<0.05, respectively). Similarly, single and double mutant strains lacking *ompP5* invaded A549 cells significantly less than the wild-type strain (p<0.0001) (Fig 2E). To further confirm P5 involvement in *H*. *influenzae* epithelial infection, we compared cell infection by *H*. *influenzae* RdKW20 wild-type and Δ*ompP5* strains. We observed P5_RdKW20_ implication in RdKW20 infection of NCI H-292 (adhesion, p<0.05; invasion, p<0.001) and A549 (adhesion, p<0.005; invasion, p<0.01) cells (Fig 2F).

Altogether, these results suggest a differential contribution of P5 and Hap to NTHi interface with the airways epithelial cells. P5 and Hap are involved in NTHi375 interplay with RPMI 2650 nasal cells. P5 seems to play a role in bacterial entry into Detroit 562 cells, and it is required for NTHi375 interplay with NCI H-292 bronchial and A549 alveolar cells, at both the adhesion and invasion levels. Hap does not participate in the NTHi-bronchial/alveolar cell interface, and the *hap* gene deficiency may favour NTHi375 interplay with Detroit 562 pharynx epithelial cells.

### P5 and Hap do not contribute to NTHi375 epithelial adhesion mediated by CEACAM1, α5 integrin or N-glycosylation

**Fig 3:**
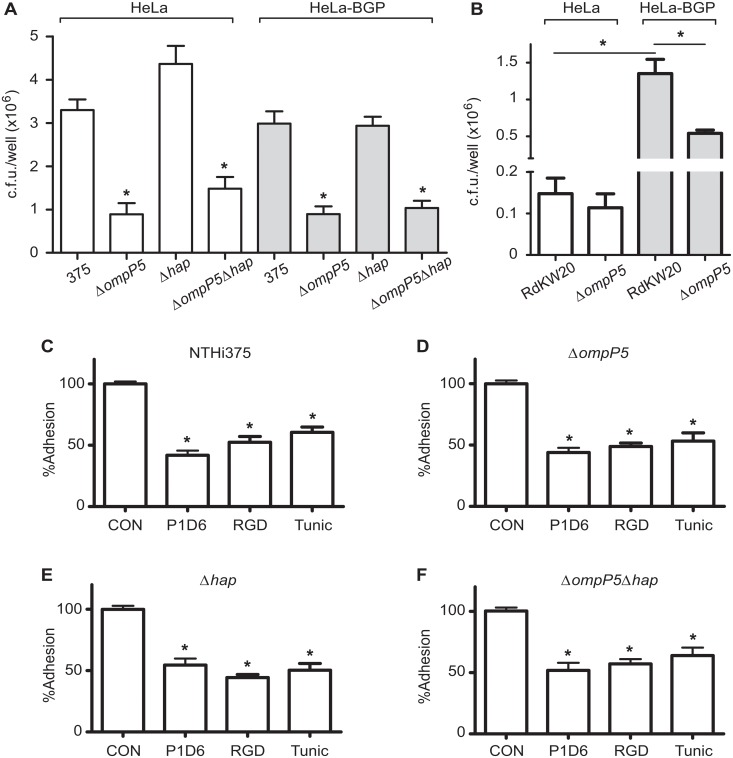
Analysis of P5 and Hap involvement in NTHi375 interaction with host cell receptors. **(A)** P5 from NTHi375 is not a ligand for CEACAM1. Effect of the *ompP5* and *hap* gene disruption on NTHi epithelial adhesion to CEACAM1, assessed by infection of HeLa-BGP and HeLa control epithelial cells. **(B)** P5 from Hi RdKW20 is a ligand for CEACAM1. Effect of the *ompP5* gene disruption on Hi RdKW20 epithelial adhesion to CEACAM1, assessed by infection of HeLa-BGP and HeLa control epithelial cells. P5 and Hap are not involved in NTHi375 interplay with α5 integrin and N-glycosylated host cell molecules. A549 cells were left untreated (CON) or were pre-incubated with blocking anti-α5 P1D6 antibody, RGD peptide, or tunicamycin, and NTHi375 **(C)**, Δ*ompP5* **(D)**, Δ*hap* **(E)** and Δ*ompP5*Δ*hap* **(F)** bacterial adhesion was determined. Data are shown as % adhesion related to control untreated cells. Experiments were performed in triplicate in at least three independent occasions (n≥9).

It has been previously reported that, depending on the strain, P5 could be a bacterial ligand for the eukaryotic receptor CEACAM1 [14]. NTHi375 adhesion assays were performed by using HeLa-BGP (biliary glycoprotein or CD66a, currently known as CEACAM1) cells [34], a HeLa derivative cell line stably expressing hCEACAM1-4L [5], and previously used to assess the impact of CEACAM1 on bacterial infections [5,39]. NTHi375 showed similar adhesion to HeLa control and HeLa-BGP cells, excluding a potential role for CEACAM1 in NTHi375 epithelial adhesion. Adhesion of NTHi375Δ*ompP5* and Δ*ompP5*Δ*hap* was lower than that displayed by the wild-type strain, to the same extent for both for HeLa (p<0.0001 and p<0.0005, respectively) and HeLa-BGP (p<0.0005) cells (Fig 3A). These results suggest that NTHi375 does not interact with CEACAM1, that P5_NTHi375_ is not likely to be a ligand for CEACAM1, and that P5, but not Hap, participates in NTHi375 adhesion to HeLa epithelial cells.

To further validate the use of HeLa-BGP cells, we took advantage of the previously shown interaction between P5_RdKW20_ and CEACAM1 [14]. As expected, Hi RdKW20 was shown to adhere better to HeLa-BGP than to HeLa cells (p<0.0001), and Hi RdKW20Δ*ompP5* showed lower adhesion to HeLa-BGP cells than the wild-type strain, (p<0.0005) (Fig 3B). Of note, Hi RdKW20Δ*ompP5* also showed to adhere better to HeLa-BGP than to HeLa cells (p<0.0001).

We have previously shown that α5β1 integrin subunit is implicated in NTHi A549 cell infection [35]. The bacterial ligand involved in this process is unknown, but it is likely to be a surface protein encompassing a RGD domain. Given that P5 and Hap do not contain a RGD domain in their respective amino acid sequences, these proteins are unlikely to be NTHi ligands for α5 integrin. As expected, NTHi375Δ*ompP5*, Δ*hap* and Δ*ompP5*Δ*hap* adhesion to A549 cells decreased in the presence of the anti-integrin α5 P1D6 blocking antibody, and of a synthetic peptide containing a RGD-sequence mimicking the physiological α5 integrin ligand fibronectin, compared to control untreated cells, same as observed for the wild-type strain (Fig 3C–3F). N-glycosylation of the α5β1 integrin receptor has been shown to be essential for association of the heterodimer subunits, and for its optimal binding to fibronectin [40]. Moreover, host cell surface glycoprotein structures may play a role in NTHi attachment to Chang epithelial cells [41]. We assessed the relevance of cell N-glycosylation on NTHi epithelial adhesion by A549 cell treatment with the N-linked glycosylation blocking agent tunicamycin. NTHi375 wild-type, Δ*ompP5*, Δ*hap* and Δ*ompP5*Δ*hap* showed a reduced adhesion to epithelial cells in the presence of tunicamycin, compared to control untreated cells. In all cases, bacterial adhesion to anti-α5 P1D6 antibody, RGD peptide or tunicamycin-treated cells was lower (p<0.0001) than to control untreated cells (Fig 3C–3F). These results suggest that NTHi375 infection of A549 cells may involve bacterial interaction with N-glycosylated residues on the host cell surface, but P5 and Hap are unlikely to participate in such interaction.

### Role of P5 and Hap in NTHi interaction with alveolar macrophages

**Fig 4:**
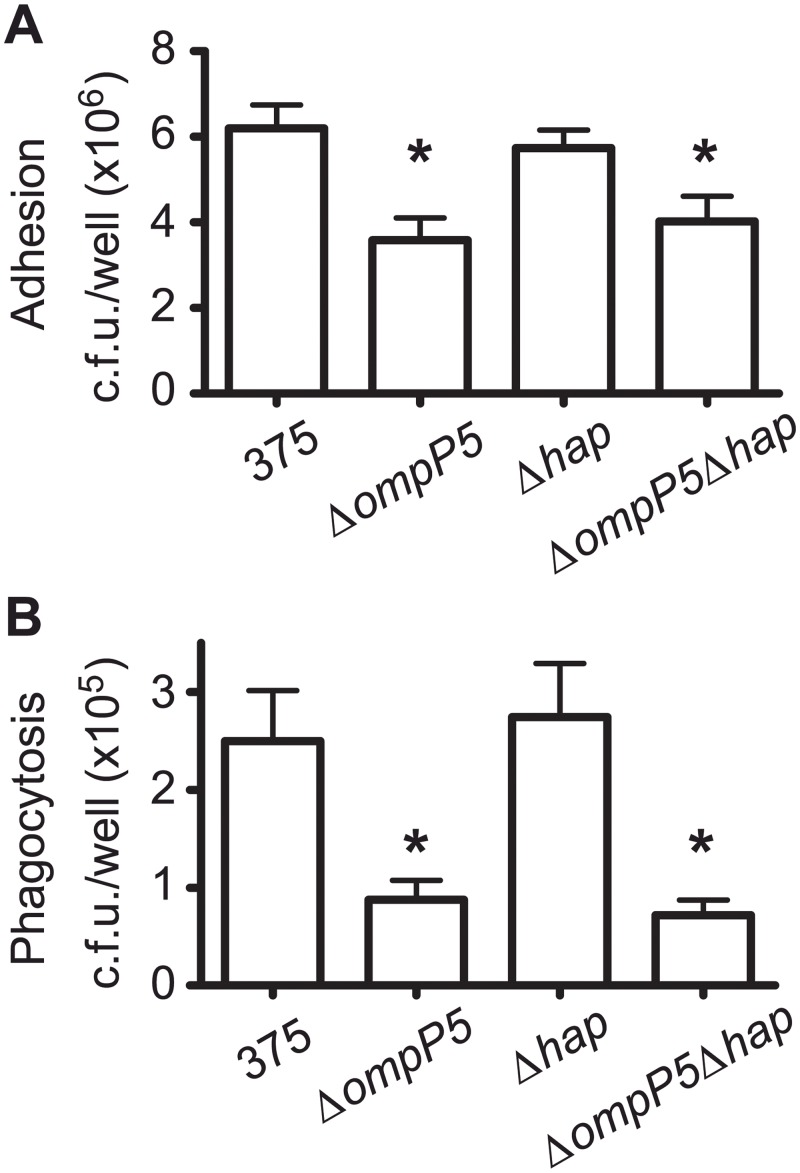
Interaction of NTHi375 mutants lacking the *ompP5* and *hap* genes with alveolar macrophages. NTHi375, Δ*ompP5*, Δ*hap* and Δ*ompP5*Δ*hap* strains were used to assess adhesion to- **(A)** and phagocytosis by- **(B)** MH-S alveolar macrophages. Experiments were performed in triplicate in at least three independent occasions (n≥9).

The lung contains alveolar macrophages which are both sentinels and the first line of defence against infection [42]. Clearance of NTHi from lungs depends on the efficiency of host phagocytes to recognise and destroy the pathogen, as we have previously described [36]. We next investigated the ability of MH-S alveolar macrophages to engulf NTHi375Δ*ompP5*, Δ*hap* and Δ*ompP5*Δ*hap* mutant strains. Adhesion of NTHi375Δ*ompP5* and Δ*ompP5*Δ*hap* to MH-S cells was lower than that displayed by the wild-type strain (p<0.005 and p<0.05, respectively). Differently, adhesion of NTHi375Δ*hap* to MH-S cells was similar to the wild-type strain (Fig 4A). In agreement with adhesion data, phagocytosis of NTHi375Δ*ompP5* and Δ*ompP5*Δ*hap* by MH-S cells was significantly lower than that displayed by the wild-type strain (p<0.01 and p<0.005, respectively), and NTHi375Δ*hap* mutant was engulfed by MH-S cells at the same level as the wild-type strain (Fig 4B). These results suggest a relevant role for P5 in NTHi recognition and engulfment by alveolar macrophages, together with a differential contribution of P5 and Hap to NTHi375 interface with this cell type.

### Heterologous expression of Hap_NTHi375_ in Hi RdKW20 shows Hap involvement in bacterial adhesion to host cells

**Fig 5:**
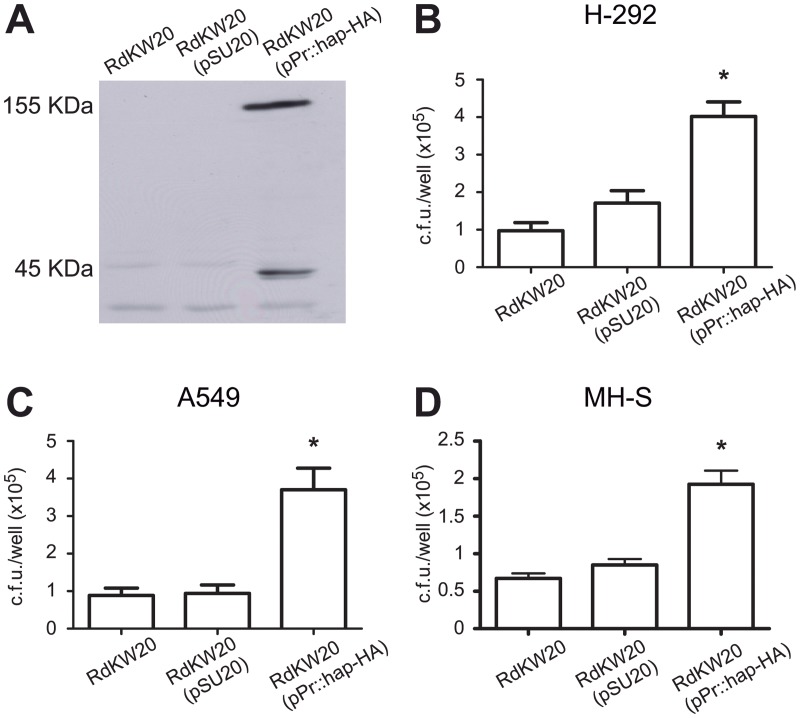
Effect of Hap_NTHi375_ expression in Hi RdKW20 adhesion to host cells. **(A)** Hap_NTHi375_ expression in Hi RdKW20. Whole cell extracts of Hi RdKW20, RdKW20 (pSU20) and RdKW20 (pSU20-*Pr*::*hap*_*NTHI375*_-HA) cultures were prepared and used to detect Hap-HA by immunoblotting with a rabbit anti-HA antibody, which reacts with Hap precursor (155 KDa) and Hap_β_ (45 KDa). Strains Hi RdKW20, RdKW20 (pSU20) and RdKW20 (pSU20-*Pr*::*hap*_*NTHI375*_-HA) were used to infect **(B)** NCI H-292, **(C)** A549, and **(D)** MH-S cells. The number of adherent bacteria per well is shown for each strain. Experiments were performed in triplicate, at least three independent times (n≥9).

Hap involvement in NTHi colonisation of the human airways has been mostly concluded from gain of function studies, based on plasmid- or chromosome expression of the *hap* gene into Hi RdKW20 [20,21,43]. Under the conditions tested in this study, the *hap* gene did not show a significant role in NTHi375 interplay with respiratory cells. Hap_NTHi375_ contains a SAAT domain (Fig A in S1 File), and shows sequence conservation on the autoproteolysis regions comprising the canonical catalytic amino acids triad and the previously established cleavage sites (Fig 1A). Therefore, this protein is likely to undergo cleavage of its passenger domain Hap_s_. To determine Hap expression and autoproteolysis for NTHi375, the *hap* gene was HA-tagged, cloned into pSU20 together with its putative promoter region, and expressed in Hi RdKW20, a strain naturally lacking a functional Hap protein due to a stop codon in the *hap* gene [44]. As expected, the 155-kDa full-length Hap_NTHi375_ protein and the 45-kDa Hap_β_ preferred cleavage product were detected in whole cell extracts by immunoblot with an anti-HA antibody (Fig 5A). Thus, according to its amino acid sequence, Hap_NTHi375_ is expressed rendering a full length precursor protein and an outer membrane translocator domain Hap_β_.

To further confirm Hap_NTHi375_ functionality, we investigated its role in bacterial adhesion to epithelial cells and alveolar macrophages, by comparing the ability of strains Hi RdKW20, Hi RdKW20 (pSU20) and Hi RdKW20 (pSU20-*Pr*::*hap*_*NTHi375*_-HA) to adhere to NCI H-292, A549 and MH-S cells. Hi RdKW20 (pSU20-*Pr*::*hap*_*NTHi375*_-HA) adhesion to the three cell types was significantly higher than that displayed by Hi RdKW20 and RdKW20 (pSU20) strains (p<0.0001, p<0.0005, p<0.0001, respectively) (Fig 5B–5D). In conclusion, the *hap*_NTHi375_ gene is likely to express an adhesive protein, as shown by its heterologous expression in the naturally *hap* deficient strain Hi RdKW20.

### Contribution of P5 and Hap to NTHi375 mouse pulmonary infection

**Fig 6:**
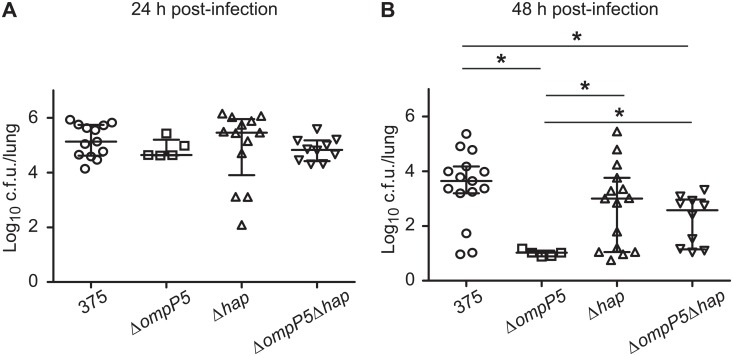
Bacterial loads in the lungs of CD1 mice infected by NTHi375 mutant strains lacking the *ompP5* and *hap* genes. Mice were infected intranasally with ~10^8^ bacteria. Bacterial counts in lungs at 24 or 48 h PI were determined. Results are reported as log_10_ c.f.u./lung. Statistical differences were seen at 48 h PI.

We have previously assessed NTHi persistence on a mouse infection model by intranasal inoculation of CD1 mice with NTHi375 [29]. In this study, we sought to determine the impact of P5 and Hap deficiency in mouse lung infection. We quantified bacterial loads for wild-type and each mutant from lung homogenates of infected mice at 24 and 48 h post-infection (PI). We recovered comparable bacterial numbers for NTHi375 wild-type, Δ*ompP5*, Δ*hap*, and Δ*ompP5*Δ*hap* strains at 24 h PI (Fig 6A). Conversely, at 48 h PI, we recovered significantly fewer bacteria for NTHi375Δ*ompP5* (p<0.001) and Δ*ompP5*Δ*hap* (p<0.05) than for the wild-type strain (Fig 6B). NTHi375Δ*hap* delivered counts indistinguishable from those obtained after infection with the wild-type strain. Of note, NTHi375Δ*ompP5*Δ*hap* rendered a significant increase in the number of bacteria recovered, when compared to NTHi375Δ*ompP5* (p<0.05). These data indicate that P5 may delay the clearance of NTHi in mouse pulmonary infection. Hap does not seem to contribute to NTHi persistence in the mouse lung, and its absence partially restores the deficiency in lung infection observed for the NTHi375Δ*ompP5* mutant strain.

## Discussion

This study delineates novel roles for P5 and Hap surface proteins in the interplay between NTHi and the respiratory system. Our systematic use of microfermenters, respiratory cultured cells and mouse respiratory infection model systems has allowed for the first time to comprehensively compare single and double P5 and Hap defective mutants generated in a pathogenic genome sequenced strain, NTHi375 [37]. Previous studies separately explored P5, Hap and their roles in NTHi-host interface, by using mutant strains made in different pathogenic strain backgrounds. The ability of NTHi1128Δ*ompP5* mutant to adhere to epithelial cell surfaces has been evaluated on A549 type II pneumocytes [10], epithelial cells in the chinchilla middle ear [9], and CHO-ICAM-1 transfected cells [18]; the ability of Hi RdKW20Δ*ompP5* to adhere to CEACAM1 has been evaluated by using CEACAM1-Fc in a receptor overlay assay [14]. Also, high throughput sequencing of transposon insertion sites pointed the importance of *ompP5* for *H*. *influenzae* RdKW20 survival in the mouse lung [13]. Although the ability of NTHiN187Δ*hap* mutant to adhere to epithelial cells has been evaluated by using Chang cells [19], most studies devoted to decipher Hap adhesive properties have been performed by expressing the *hap* gene in strain DB117, a Hi RdKW20 derivative deficient in *rec1* [20,43].

Our results indicate that P5 is involved in several aspects of the NTHi-host interplay. Although not required for biofilm growth and with a moderate role in NTHi interaction with upper airways cells, NTHi375Δ*ompP5* was impaired in terms of adhesion and entry into non-phagocytic bronchial and alveolar epithelial cells, and in adhesion to- and uptake by professional phagocytes. Our results suggest that P5 may be a bacterial ligand for host receptor(s) present on the surface of these cell types, although CEACAM1 and α5 integrin are unlikely to be P5 receptors for NTHi375. CEACAM1 has been previously identified as a receptor for several *H*. *influenzae* strains [45], targeted by P5 or by bacterial ligands alternative to P5 [14]. This was not the case for NTHi375, despite the presence or absence of the *ompP5* gene. P5 extracellular loop domains display sequence variability (Table A in S1 File) [6,46], which could account for the observed phenotypic heterogeneity among isolates. Of note, our results confirmed that P5_RdKW20_ is likely to bind CEACAM1, together with additional currently unknown bacterial ligands [14], and a panel of genome sequenced NTHi clinical isolates tested in terms of interaction with CEACAM1, has rendered a heterogeneous behavior among strains (B. Euba, personal communication), therefore validating the use of HeLa-BGP cells, and supporting the notion of a significant variability among isolates. Previous evidence suggests that P5 could be a bacterial ligand for ICAM-1 [18], and ICAM-1 is a heavily N-glycosylated transmembrane protein [47]. Given that tunicamycin reduced NTHi adhesion to alveolar epithelial cells for both the wild-type and the Δ*ompP5* mutant strains, NTHi375 may interact with N-glycosylated molecules at the host cell surface, but P5 is unlikely to be a ligand involved in such interaction. We speculate that the significant *in vivo* clearance observed for NTHi375Δ*ompP5* in our respiratory infection mouse model could be a consequence of this mutant impairment to firmly attach to host cell surfaces. The fast lung clearance displayed by NTHi375Δ*ompP5* is in agreement with previous observations for intranasal infection of Hi RdKW20 lacking the *ompP5* gene [13].

Hap seemed to play a limited role in most aspects analysed in this work. Hap_NTHi375_-HA detection in whole-cell extracts when expressed in the *hap* naturally lacking strain RdKW20, and the gain of cell adhesive function by RdKW20 when transformed with pSU20-*Pr*::*hap*_*NTHi375*_-HA, supported the functionality of the *hap*_*NTHi375*_ gene. However, NTHi375Δ*hap* infected bronchial and alveolar epithelial cells, and alveolar macrophages at similar levels than the wild-type strain. We speculate that *hap* deficiency could be compensated by other NTHi surface molecules with potentially redundant function, therefore masking Hap-driven clear cut phenotypes. Hap autoproteolytic activity has been shown to be inhibited by SLPI, which protects the respiratory epithelium from injury due to neutrophil elastase and other proteases involved in acute inflammation [48]. During natural infection, inhibition of Hap autoproteolysis presumably facilitates *H*. *influenzae* colonization of the respiratory mucosa, while release of Hap_s_ may result in dispersal and migration from the site of infection. The *hap* gene deficiency would eliminate this predicted Hap involvement in colonisation facilitated by SLPI-dependent inhibition of Hap autoproteolysis. Based on this notion, NTHi375Δ*hap* would lack one of NTHi colonising predicted strategies. However, the results obtained in this study do not support such hypothesis, given that NTHi375Δ*hap* mouse lung infection was comparable to that observed for the wild-type strain. Of note, NTHi375Δ*ompP5*Δ*hap* double mutant lung bacterial load after a 48 h infection was higher than that obtained for NTHi375Δ*ompP5*.

We acknowledge that extrapolation of the results obtained on cultured human cell lines to tissue locations *in vivo* should be considered with caution. Also, some of the phenotypes obtained for the *ompP5* gene agree with those previously shown for different strain backgrounds, supporting the results presented in this study; moreover, the gain of adhesive function by heterologous expression of the *hap* gene in the Rd KW20 naturally *hap* deficient strain allowed us to assign an adhesive role to Hap_NTHi375_. In conclusion, this study presents a comparative analysis of NTHi-host interaction through cells representing different anatomical locations of the respiratory tract, frames the relative contribution of two major bacterial surface proteins, P5 and Hap, to this interplay, and extends our understanding of the colonisation and infection process by NTHi. We acknowledge that, considering the observed heterogeneity among NTHi strains [4], further systematic correlation between large repertoires of well characterised NTHi isolates and wide panels of relevant phenotypic traits, together with a detailed characterisation of cell surface receptors, will contribute to unravel key bacterial and host cell elements for the NTHi-host interplay. Interference strategies designed to abolish such interplay would limit NTHi adaptation to the human respiratory tract, therefore preventing colonisation and/or pathogenicity.

## Supporting Information

S1 File Table A, Level of conservation of P5NTHi375 and HapNTHi375. Length and percentages of identity, similarity and gaps for P5_NTHi375_ and Hap_NTHi375_, compared to P5 and Hap orthologous proteins from NTHi strains listed below (BLASTp results). Fig A, Multiple sequence alignment for 311 amino acids at the C-terminal region of Hap_s-NTHi375_ domain, corresponding to the SAAT domain. Alignment was performed in Muscle [49], by using all NCBI available NTHi orthologous proteins.
(PDF)
