# Supplementary material for: Relative Contribution of P5 and Hap Surface Proteins to Nontypable Haemophilus influenzae Interplay with the Host Upper and Lower Airways
Source: PLoS One. 2015 Apr 20;10(4):e0123154. doi: 10.1371/journal.pone.0123154 (PMC4403991; doi:10.1371/journal.pone.0123154)
Supplement: S1 File — Table A, Level of conservation of P5NTHi375 and HapNTHi375. Length and percentages of identity, similarity and gaps for P5NTHi375 and HapNTHi375, compared to P5 and Hap orthologous proteins from NTHi strains listed below (BLASTp results). Fig A, Multiple sequence alignment for 311 amino acids at the C-terminal region of Haps-NTHi375 domain, corresponding to the SAAT domain. Alignment was performed in Muscle [49], by using all NCBI available NTHi orthologous proteins. (PDF) [file pone.0123154.s001.pdf]

Supporting information

Table A. Level of conservation of $P5_{NTHi375}$ and $Hap_{NTHi375}$. Length and percentages of identity, similarity and gaps for $P5_{NTHi375}$ and $Hap_{NTHi375}$, compared to P5 and Hap orthologous proteins from NTHi strains listed below (BLASTp results).

	**$P5_{NTHi375}$**				**$Hap_{NTHi375}$**			
	**Length**	**%identity**	**%similarity**	**%gaps**	**Length**	**%identity**	**%similarity**	**%gaps**
NTHi KR494	347	96.8	98.3	0	1433	72.9	79.6	6.6
NTHi R2846	359	86.7	90.6	3.9	1385	69.4	77	9.2
NTHi R2866	358	87.2	90.8	3.6	1404	80.8	86	1.6
NTHi 10810	353	94.4	95.5	2.3	1400	78.2	84.8	2.3
NTHi 86028NP	359	86.9	90.6	3.9	1392	75.1	82.7	3.9
NTHi F3031	348	92.6	95.4	1.4	ND	ND	ND	ND
NTHi F3047	348	92.6	95.4	1.4	ND	ND	ND	ND
NTHi RdKW20	353	94.6	96.3	1.7	-[b]	-	-	-
NTHi PittAA	355	93.5	95.8	2.3	-	-	-	-
NTHi PittHH	358	87.2	90.8	3.1	-	-	-	-
gb|AAN37924.1|	ND[a]	ND	ND	ND	1391	88.7	92.8	0.4
gb|AAB03707.1|	ND	ND	ND	ND	1393	75.4	83.6	2.4
gb|AAN37923.1|	ND	ND	ND	ND	1436	75.8	82.9	5
ref|WP_005686673.1|	ND	ND	ND	ND	1379	78.3	85.3	2.3
ref|WP_005692206.1|	ND	ND	ND	ND	1391	74.9	83	2.1
ref|WP_005653405.1|	ND	ND	ND	ND	1401	75.5	83.1	2.5

ND[a]: not determined

-[b]: the *hap* gene contains a stop codon generating a frameshift

Figure A. Multiple sequence alignment for 311 amino acids at the C-terminal region of Hap$_{s\text{-}NTHi375}$ domain, corresponding to the SAAT domain. Alignment was performed in Muscle [48], by using all NCBI available NTHi orthologous proteins.

```
NTHi Hi375jt_Hap      -TICTRSDWTGLTTCKTVNLTDKKVIDSIPTTQINGSINLTDNATVNINGLAKLNGNVTLIDHSQFTLSNNATQIGNIKLSNHANATVDNA-788
gb|AAN37924.1|        -TICTRSDWTGLTTCKTVDLTDKKVINSIPTTQINGSINLTDNATVNIHGLAKLNGNVTLIDHSQFTLSNNATQTGNIKLSNHANATVDNA-787
gb|AAB03707.1|        -TICTRSDWTGLTTCQKVDLTDTKVINSIPKTQINGSINLTDNATANVKGLAKLNGNVTLTNHSQFTLSNNATQIGNIRLSDNSTATVDNA-785
ref|WP_005686673.1|   -TICTRSDWTGLTTCKTENLTDTKVINSIPTTQINGSINLTDNATANVKGLAKLNGNVTLTNHSQFTLSNNATQIGNIKLSNHANATVDNA-777
ref|WP_005692206.1|   -TICTRSDWTGLTTCKTENLTDTKVINSIPTTQINGSINLTDNATANVKGLAKLNGNVTLTNHSQFTLSNNATQIGNIKLSNHANATVDNA-782
NTHi 86-028NP         -TICTRSDWTGLTTCKTENLTDTKVINSIPTTQINGSINLTDNATANVKGLAKLNGNVTLTNHSQFTLSNNATQIGNIRLSDNSTATVDNA-784
ref|WP_005653405.1|   -TICTRSDWTGLTTCKTENLTDTKVINSIPTTQINGSINLTDNATANVKGLAKLNGNVTLTNHSQFTLSNNATQTGNIKLSNHANATVDNA-792
NTHi KR494            -TICTRSDWTGLTTCKTVDLNNNAVIASIPITQINGSINLTDNATTNVKGLAKLNGNVTLTNHSQFTLSNNATQIGNIKLSNHANATVNNA-786
NTHi R2846            -TICTRSDWTGLTECKSENLTEQKTIDSLPRTKVNGNVHLMNSASAVASGLVDLIGNVTLTQNSQFTLSHNATQTGSLSLVNQANATVNNA-786
NTHi R2866            -TICTRSDWTGLTTCQKVDLTDTKVINSIPTTQINGSINLTDNATANVKGLAKLNGNVTLTNHSQFTLSNNATQIGNIKLSNHANATVDNA-794
NTHi 10810            -TICTRSDWTGLTTCKTVDLNNNAVIASIPITQINGSINLTDNATANIHGLAKLNGNVTLINHSQFTLSNNATQIGNIKLSNHANATVNNA-797

NTHi Hi375jt_Hap      -TLNG---------------------------------------NVNLTDSAQFSLKNSHFSHQIQGDKGTTVTLENATWTMPSDATLQNL-839
gb|AAN37924.1|        -NLNG---------------------------------------NVNLMDSAQFSLKNSHFSHQIQGGEDTTVMLENATWTMPSDTTLQNL-838
gb|AAB03707.1|        -NLNG---------------------------------------NVHLTDSAQFSLKNSHFSHQIQGDKGTTVTLENATWTMPSDTTLQNL-836
ref|WP_005686673.1|   -TLTG---------------------------------------NVYLTDSARFSLKNSHFSHQIQGDKGTTVTLENSTWTMPSDTTLQNL-828
ref|WP_005692206.1|   -TLTG--------------------------------------DVNLTDSAQFYLKNSHFSHQIQGDKDTTVTLENATWTMPSDTTLQNL-833
NTHi 86-028NP         -NLNG---------------------------------------NVHLTNSAQFYLKNSHLSHQIQGDKGTTVTLENATWTMPSDTTLQNL-835
ref|WP_005653405.1|   -NLNG---------------------------------------NVHLTDSAQFYLKNSHFSHQIQGDKDTTVTLENATWTMPSDTTLQNL-843
NTHi KR494            -TLTGDVNLADTSHFTLNNHATQIGTISLHQQAQATVDNANLNGNVHLTDSAQFYLKNSHFSHQIQGDKGTTVTLENATWTMPSDTTLQNL-876
NTHi R2846            -TLTGDVNLADTSRFTLSNQATQIGTISLHQQAQATVDNANLNGNVHLTDSAQFSLKNSHFSHQIQGDKDTTVTLENATWTMPSDTTLQNL-876
NTHi R2866            -TLTG--------------------------------------DVNLTDSAQFYLKNSHFSHQIQGDKGTTVMLENATWTMPGDTTLQNL-845
NTHi 10810            -TLMG---------------------------------------NVNLTDSAQFYLKNSHFSHQIQGDKDTTVTLENATWTMPSDTTLQNL-848

NTHi Hi375jt_Hap      -TLNNSTVTLNSAYSASSNNAP-RHRRSLETETTPTSAEHRFNTLTVNGKLSGQGTFQFTSSLFGYKSDKLKLSNDAEGDYTLSVRNTGKE-928
gb|AAN37924.1|        -TLNNSTVTLNSAYSAISNNAPRRRRRSLETETTPTSAEHRFNTLTVNGKLSGQGTFQFTSSLFGYKSDKLKLSNDAEGDYTLSVRNTGKE-928
gb|AAB03707.1|        -TLNNSTITLNSAYSASSNNTP--RRRSLETETTPTSAEHRFNTLTVNGKLSGQGTFQFTSSLFGYKSDKLKLSNDAEGDYILSVRNTGKE-924
ref|WP_005686673.1|   -TLNNSTVTLNSAYSASSNNAPRRRRRSLDTETTPTSAEHRFNTLTVNGKLSGQGTFQFTSSLFGYKSDKLKLSNDAEGDYTLSVRNTGKE-918
ref|WP_005692206.1|   -TLNNSTVTLNSAYSATTNNTPRRRRRSLETETTPTSAEHRFNTLTVNGKLSGQGTFQFTSSLFGYKSDKLKLSKDAEGTYKLAVRNTGKE-923
NTHi 86-028NP         -TLNNSTVTLNSAYSASPNNTPRRRRRSLETETTPTSAEHRFNTLTVNGKLSGQGTFQFTSSLFGYKSDKLKLSNDAEGDYILSVRNTGKE-925
ref|WP_005653405.1|   -TLNNSTVTLNSAYSASSNNTPRRRRRSLETETTPTSAEHRFNTLTVNGTLKGKGTFQFTSSLFGYKSDKLKLSNDAEGDYTLSVRDTGKE-933
NTHi KR494            -TLNNSTVTLNSAYSASSNNTPRRRRRSLETETTPTSAEHRFNTLTVNGKLSGQGTFQFTSSLFGYKSDKLKLSNHAEGDYTLAVRNTGKE-966
NTHi R2846            -TLNNSTVTLNSAYSASPNNAPRRRRRSLETETTPTSAEHRFNTLTVNGKLSGQGTFQFTSSLFGYKSDKLKLSNDAEGDYILAVRDTGKE-966
NTHi R2866            -TLNNSTVTLNSAYSASSNNTPRRRRRSLETETTPTSAEHRFNTLTVNGKLSGQGTFQFTSSLFGYKSDKLKLSNDAEGDYILAVRDTGKE-935
NTHi 10810            -TLNNSTVTLNSAYSASSNNAP-RHRRSLETETTPTSAEHRFNTLTVNGKLRGKGTFQFTSSLFGYKSDKLKLSNDAEGDYTLSVRNTGKE-937

NTHi Hi375jt_Hap      -PEALEQLTLVESKDNQPLSDKLKFTLENDHVDAGALRYKLVKNKGEFRLHNPIKEQELRNDLVRAEQAERTLEAKQVEQTAKTQTSKARV-1018
gb|AAN37924.1|        -PVTFGQLTLVESKDNKPLSDKLTFTLENDHVDAGALRYKLVKNDGEFRLHNPIKEQELRSDLVRAEQAERTLEAKQVEQTAKTQTSKARV-1018
gb|AAB03707.1|        -PETLEQLTLVESKDNQPLSDKLKFTLENDHVDAGALRYKLVKNDGEFRLHNPIKEQELHNDLVRAEQAERTLEAKQVEPTAKTQTGEPKV-1014
ref|WP_005686673.1|   -PEALEQLTLVESKDNQPLSDKLTFTLENDHVDAGALRYKLVKNKGEFRLHNPIKEQELRNDLVRAEQAEQTLEAKLAELTDKKQKGEAKV-1008
ref|WP_005692206.1|   -PVTLEQLTLIESLDNQPLSDKLKFTLENDHVDAGALRYRLVKNNGEFRLHNPIKEQELHNDLVRAEQAERTLEAKQVEPTAKTQTGEPKV-1013
86-028NP              -PETLEQLTLVESKDNQPLSDKLTFTLENDHVDAGALRYVLVKNNGEFRLHNPIKEQELHNDLVRAEQAEQTLEAKQVEPTAETQTSKAKV-1015
ref|WP_005653405.1|   -PETLEQLTLIEGLDNQRLSDKLKFTLENDHVDAGALRYRLVKNNGEFRLHNPIKEQELHNDLVKAEQAERTLEAKQAELTAETQTGKAKV-1023
NTHi KR494            -PVTLEQLTLIESLDNQPLSDKLTFTLENDHVDAGAWRYKLVKKNGEFRLYNPIKEQELTDNLANAEQREQALKEKQDELTAKKQKPEAKV-1056
NTHi R2846            -PKTLEHLTLVESKDNKPLSDKLTFTLENKHVDAGALRYELVKKNGEFRLHNPIKEQELHDDLVRAEQTERTLEAKQAELTDKKQKTKAKV-1056
NTHi R2866            -PETLGQLTLVESKDNQPLSDKLKFTLENDHVDAGALRYKLVKNNGEFRLHNPIKEQELHNDLVRAEQAERTLEAKQVEPTAKTQTGEPKV-1025
NTHi 10810            -PEALEQLTLVESKDNKPLSDKLKFTLENDHVDAGALRYELVKNNGEFRLHNPIKEQELHNDLVRAEQAERTLEAKQVEQTAETQTSQARV-1027

NTHi Hi375jt_Hap      -RSR---RAVFSDPLPAQSLLNALEAKQA-LTTETQTSKAK--KVRSKRAARE-FSDTLPDQ----ILQAALEVIDAQQQVKKEPQTQEEE-1097
gb|AAN37924.1|        -RSR---RAVFSDPLPAQSLLKALEAKQA-LTTETQTSKAK--KVRSKRAARE-FSDTLPDQ----ILQAALEVIDAQQQVKKEPQTQEEE-1097
gb|AAB03707.1|        -RSRRAARAAFPDTLPDQSLLNALEAKQAELTAETQKSKAKTKKVRSKRAV---FSDPLLDQS-LFALEAALEVIDAPQQSEKDRLAQE-E-1099
ref|WP_005686673.1|   -RLR---RAVLSDTPSAQSLLNVLEAKQVELTAEKQPSKAKTKKVRSKRALREAFSDTQPDQIPLNVLKAALEVIDAQQQV-----------1085
ref|WP_005692206.1|   -RSRRAARAAFPDTLPDQSLLNALEAKQAELTAETQKSKAKTKKVRSKRAV---FSDPPLDS--LFALEAALEVIDAPQQSEKDRLAQE-E-1097
NTHi 86-028NP         -RLR---RAVLPDTLPAQSLLNALEAKQAEPNAKTQKSKAKTKKARSKRALRAVFSDPPPDQSQLDVLKAALEVINAQPQVGKERQAQE---1100
ref|WP_005653405.1|   -RLR---RAAFSDTSPDQSLLNSLEAKQVELTAETQKSKAKTKKARSKRAL---FFDTPPDLSQLKVLEVKLEAINAQQQAEKERQAQEEE-1107
NTHi KR494            -RSK---RAAFSDTSPDQSQLNALEAKQAELTAETQKSKAKTKKGRSKRAV---FSDPPLDQSLLNVLQAALEVIDVQQQVEKERKAQE-Q-1139
NTHi R2846            -RSK---RAVFFDTLP------------------------------------------PPDQSQLNILRAGLEVINAQQQV-----------1091
NTHi R2866            -RSRRAARAAFPDTLPDQSLLNALEAKQA-LTAEKQKSKAK--KGRSKRAARA-FSDTPLDLSQ-KVLEVKLEAINAQPQVGKERQTQEEQ-1110
NTHi 10810            -RSR---RAVFSDTPSAQSLLNALEAKQA-LTAEKQKSKAKTKKARSKRAL---FFDTPPDQRQLDVLKAALEVINAQPQVGKERQAQE---1108
```
